# Angiographic Findings and Post–Percutaneous Coronary Intervention Fractional Flow Reserve

**DOI:** 10.1001/jamanetworkopen.2024.18072

**Published:** 2024-06-21

**Authors:** Jinlong Zhang, Doyeon Hwang, Seokhun Yang, Xinyang Hu, Joo Myung Lee, Chang-Wook Nam, Eun-Seok Shin, Joon-Hyung Doh, Masahiro Hoshino, Rikuta Hamaya, Yoshihisa Kanaji, Tadashi Murai, Jun-Jie Zhang, Fei Ye, Xiaobo Li, Zhen Ge, Shao-Liang Chen, Tsunekazu Kakuta, Jian’an Wang, Bon-Kwon Koo

**Affiliations:** 1 Department of Cardiology, Second Affiliated Hospital of Zhejiang University School of Medicine, State Key Laboratory of Transvascular Implantation Devices, Hangzhou, China; 2 Department of Internal Medicine and Cardiovascular Center, Seoul National University Hospital, Seoul, Korea; 3 Division of Cardiology, Department of Internal Medicine, Heart Vascular Stroke Institute, Samsung Medical Center, Sungkyunkwan University School of Medicine, Seoul, Korea; 4 Department of Cardiology, Keimyung University Dongsan Medical Center, Daegu, Korea; 5 Department of Cardiology, Ulsan University Hospital, University of Ulsan College of Medicine, Ulsan, Korea; 6 Department of Cardiology, Inje University Ilsan Paik Hospital, Goyang, Korea; 7 Division of Cardiovascular Medicine, Tsuchiura Kyodo General Hospital, Ibaraki, Japan; 8 Division of Cardiology, Nanjing First Hospital, Nanjing Medical University, Nanjing, China

## Abstract

**Importance:**

The associations between angiographic findings and post–percutaneous coronary intervention (PCI) fractional flow reserve (FFR) and their clinical relevance according to residual functional disease burden have not been thoroughly investigated.

**Objectives:**

To evaluate the association of angiographic and physiologic parameters according to residual functional disease burden after drug-eluting stent implantation.

**Design, Setting, and Participants:**

This cohort study population was from the International Post-PCI FFR registry, which incorporated 4 registries from Korea, China, and Japan. Patients who underwent angiographically successful second-generation drug-eluting stent implantation and post-PCI FFR measurement were included in the analysis. The patients were divided into 3 groups according to the residual disease burden (post-PCI FFR ≤0.80 [residual ischemia], 0.81-0.86 [suboptimal], and >0.86 [optimal]). The data were collected from August 23, 2018, to June 11, 2019, and the current analysis was performed from January 11, 2022, to October 7, 2023.

**Exposures:**

Angiographic parameters and post-PCI FFR.

**Main Outcomes and Measures:**

The primary outcome was target vessel failure (TVF), defined as a composite of cardiac death, target vessel–related myocardial infarction, and target vessel revascularization (TVR) at 2 years.

**Results:**

In this cohort of 2147 patients, the mean (SD) age was 64.3 (10.0) years, and 1644 patients (76.6%) were men. Based on the post-PCI physiologic status, 269 patients (12.5%) had residual ischemia, 551 (25.7%) had suboptimal results, and 1327 (61.8%) had optimal results. Angiographic parameters had poor correlations with post-PCI FFR (*r* < 0.20). Post-PCI FFR was isolated from all angiographic parameters in the unsupervised hierarchical cluster analysis. Post-PCI FFR was associated with the occurrence of TVF (adjusted hazard ratio [AHR] per post-PCI FFR 0.01 increase, 0.94 [95% CI, 0.92-0.97]; *P* < .001), but angiographic parameters were not. The residual ischemia group had a significantly higher rate of TVF than the suboptimal group (AHR, 1.75 [95% CI, 1.08-2.83]; *P* = .02) and the optimal group (AHR, 2.94 [95% CI, 1.82-4.73]; *P* < .001). The TVR in the residual ischemia group was predominantly associated with TVR in the nonstented segment (14 [53.8%]), unlike the other 2 groups (3 [10.0%] in the suboptimal group and 13 [30.2%] in the optimal group).

**Conclusions and Relevance:**

In this cohort study of the International Post-PCI FFR registry, a low degree of associations were observed between angiographic and physiologic parameters after PCI. Post-PCI FFR, unlike angiographic parameters, was associated with clinical events and the distribution of clinical events. The current study supports the use of post-PCI FFR as a procedural quality metric and further prospective study is warranted.

## Key Points

**Question:**

Are there associations of angiographic findings and post–percutaneous coronary intervention (PCI) fractional flow reserve (FFR) and residual functional disease burden, and are they associated with cardiac outcomes?

**Findings:**

In this cohort study of 2147 patients from the International Post-PCI FFR registry, poor associations were observed between angiographic and physiologic parameters after PCI. Post-PCI FFR, unlike angiographic parameters, was associated with clinical events and the distribution of clinical events.

**Meaning:**

These findings suggest that angiographic assessment of post-PCI state may not provide relevant physiologic and clinical insights after PCI, but post-PCI physiologic assessment can offer valuable information about future clinical events and their distribution.

## Teaser

This cohort study evaluates the association of angiographic findings and post–percutaneous coronary intervention (PCI) physiologic parameters according to residual functional disease burden among patients undergoing second-generation drug-eluting stent implantation.

## Introduction

Percutaneous coronary intervention (PCI) is a standard treatment option for coronary artery disease.^1,2^ Even though ischemia-guided PCI is endorsed by class IA recommendation, adverse clinical events still occur in approximately 10% of patients after the procedure.^1,2,3,4^ Traditionally, angiographically successful PCI has been defined as a minimum stenosis diameter reduction to less than 20%.^5^ Recent studies demonstrated the clinical relevance of invasive physiologic assessment after PCI—post-PCI fractional flow reserve (FFR)^6,7,8,9,10,11,12,13^—and its association with the risk of clinical events after PCI.^14,15,16^ However, the associations between angiographic findings and post-PCI FFR and their clinical relevance according to residual functional disease burden have not been fully evaluated. In this regard, we evaluated whether angiographic and post-PCI physiologic parameters according to residual functional disease burden after second-generation drug-eluting stent (DES) implantation were associated with cardiac outcomes.

## Methods

### Study Population

This cohort study evaluated a population from the International Post-PCI FFR registry,^17^ which included 4 registries from Korea, China, and Japan. All patients underwent angiographically successful PCI with second-generation DES and obtained post-PCI FFR measures. The study population has been described previously.^11^ Briefly, a standardized spreadsheet with standard definitions of each variable was requested from the principal investigators of each registry, the COE-PERSPECTIVE (Influence of FFR on the Clinical Outcome After Percutaneous Coronary Intervention) registry,^18^ the DKCRUSH (Double Kissing Crush) VII registry,^19^ the institutional registry of Tsuchiura Kyodo General Hospital, Ibaraki, Japan,^20^ and the 3V-FFR-FRIENDS (Three-Vessel Fractional Flow Reserve for the Assessment of Total Stenosis Burden and Its Clinical Impact in Patients With Coronary Artery Disease) registry.^21^ A central monitoring team at Seoul National University Hospital (J.Z. and S.Y.) double-checked all transferred data and raised queries as needed. Among a total of 2200 patients, the present study analyzed 2147 who had complete clinical, preangiographic, and postangiographic parameters and post-PCI FFR data. A representative vessel of each patient was defined as the vessel with the lowest post-PCI FFR value for the analysis in patients with multivessel interrogation. The study protocol was approved by the ethics committee of Seoul National University Hospital and conducted according to the principles of the Declaration of Helsinki.^22^ The need for patient informed consent was waived due to the retrospective nature of the study. This study followed the Strengthening the Reporting of Observational Studies in Epidemiology (STROBE) reporting guideline.

### Coronary Angiography and FFR

Invasive coronary angiography was performed according to standard techniques. Quantitative coronary angiography was performed at each core laboratory using a validated software program with optimal projections (CAAS II [Pie Medical] and QAngio XA [Medis Medical Imaging Systems BV]).^12^ Lesion length, reference diameter, minimum lumen diameter (MLD), and percentage diameter stenosis before and after PCI were estimated. Coronary revascularization was performed following the standard techniques with second-generation DES.^2^ The post-PCI quantitative coronary analysis was performed at the location where the stent was implanted. During PCI, the type of DES, stenting techniques, and use of additional imaging devices, such as intravascular ultrasonography or optical coherence tomography, were at the operating physician’s discretion.

The FFR measurements were performed using standard techniques.^23^ A pressure sensor guide wire was equalized to aortic pressure through a guiding catheter ranging from 5F to 7F and subsequently positioned at the distal segment of the target vessel. Hyperemic agents, such as adenosine, adenosine triphosphate, papaverine, and nicorandil, were used to induce hyperemia. The patients were divided into 3 groups based on post-PCI physiologic status using the cutoff values for post-PCI FFR of 0.80 and 0.86, according to previous studies.^3,4,13^ A recent large meta-analysis reported that post-PCI FFR of 0.86 was identified as the most reliable predictive factor associated with freedom from clinical events. Based on the post-PCI physiologic status, the residual ischemia group was defined as patients with a post-PCI FFR of 0.80 or less; the optimal group, patients with a post-PCI FFR of greater than 0.86; and the suboptimal group, patients with a post-PCI FFR of 0.81 to 0.86.

### Primary Outcomes and Definitions

The primary clinical outcome was target vessel failure (TVF), a composite of cardiac death, target vessel–related myocardial infarction, and target vessel revascularization (TVR) at 2 years. All deaths were considered cardiac in origin unless a noncardiac reason was indicated. The myocardial infarction was defined by the Fourth Universal Definition of Myocardial Infarction,^24^ and only spontaneous myocardial infarction was included in the analysis. Clinically driven revascularization was defined as repeat revascularization in the presence of diameter stenosis of at least 50% with at least 1 of the following: (1) recurrence of anginal symptoms; (2) positive noninvasive test result; (3) positive invasive physiologic test result; or (4) presence of diameter stenosis of at least 70%, even in the absence of other criteria. The target lesion included a 5-mm margin proximal and distal to the stent and the stent itself.

### Statistical Analysis

The data were collected from August 23, 2018, to June 11, 2019, and the present analysis was performed from January 11, 2022, to October 7, 2023. Continuous variables are presented as means (SDs) and categorical variables as numbers and relative frequencies. The χ^2^ test was used to evaluate associations between categorical variables, and an analysis of variance was performed to compare continuous variables. The Bonferroni correction was used for pairwise comparisons. Pearson correlation coefficient was calculated to assess the linear association between variables. An unsupervised hierarchical cluster analysis was used to cluster similar relevant features. Briefly, hierarchical clustering is one of the algorithms from unsupervised machine learning, which is used to group similar objects, and the objects in each cluster are broadly similar. To investigate the association of each post-PCI parameter with the outcome, a multivariable Cox proportional hazards regression model was used to calculate the adjusted hazard ratio (AHR) and its 95% CI with covariables that were considered clinically reliable or associated with clinical outcomes. The cumulative incidence of clinical event rates was presented based on Kaplan-Meier censoring estimates. A log-rank test was used to compare the cumulative incidence of clinical events. All probability values were 2 sided, and *P* < .05 was considered statistically significant. The statistical packages SPSS, version 23.0 (IBM Corp) and R, version 4.1.2 (R Project for Statistical Computing) were used for statistical analysis.

## Results

### Baseline Characteristics

Of 2147 patients, the mean (SD) age was 64.3 (10.0) years; 1644 (76.6%) were men and 503 (23.4%) were women. Racial and ethnic data were not collected. Based on the post-PCI physiologic status, 1327 patients (61.8%) were in the optimal group, 551 (25.7%) were in the suboptimal group, and 269 (12.5%) were in the residual ischemia group. The baseline clinical and procedural characteristics are shown in the Table. Patients in the residual ischemia group had a higher proportion of men and prevalence of cardiovascular risk factors (Table).

**Table.** Baseline Patient and Lesion Characteristics

	Outcome group^a^				*P* value^b^
	All (N = 2147)	Optimal (n = 1327)	Suboptimal (n = 551)	Residual ischemia (n = 269)	
Age, mean (SD), y	64.3 (10.0)	64.7 (9.8)^c^	63.9 (10.4)	63.4 (10.1)^d^	.07
Sex					
Female	503 (23.4)	342 (25.8)	115 (20.9)	46 (17.1)	.002
Male	1644 (76.6)	985 (74.2)	436 (79.1)	223 (82.9)	
Acute coronary syndrome	1111 (51.7)	746 (56.2)^c^^,^^e^	256 (46.5)^d^	109 (40.5)^d^	<.001
Diabetes	723 (33.7)	423 (31.9)^c^	191 (34.7)	109 (40.5)^d^	.02
Hypertension	1450 (67.5)	915 (69.0)	365 (66.2)	170 (63.2)	.14
Hypercholesteremia	1071 (49.9)	629 (47.4)^c^	288 (52.3)	154 (57.2)^d^	.006
Smoking	642 (29.9)	391 (29.5)	153 (27.8)^c^	98 (36.4)^e^	.03
Left ventricular ejection fraction, mean (SD), %	62.1 (8.4)	62.2 (8.2)	62.0 (8.6)	61.9 (8.5)	.93
Lesion characteristics					
Left anterior descending artery	1510 (70.3)	807 (60.8)^c^^,^^e^	474 (86.0)^d^	229 (85.1)^d^	<.001
Lesion length, mean (SD), mm	24.6 (14.3)	24.9 (14.5)	24.1 (14.1)	23.7 (13.8)	.27
Reference diameter, mean (SD), mm	2.88 (0.51)	2.94 (0.52)^c^^,^^e^	2.79 (0.49)^d^	2.75 (0.49)^d^	<.001
Pre-PCI MLD, mean (SD), mm	1.07 (0.46)	1.10 (0.48)^c^	1.05 (0.43)	0.99 (0.39)^d^	.001
Post-PCI MLD, mean (SD), mm	2.74 (0.47)	2.79 (0.47)^c^^,^^e^	2.67 (0.46)^d^	2.62 (0.48)^d^	<.001
Pre-PCI DS, mean (SD), %	62.7 (14.4)	62.7 (14.7)	62.4 (14.2)	63.8 (13.1)	.41
Post-PCI DS, mean (SD), %	9.3 (7.1)	8.9 (6.9)^c^	9.6 (7.1)	10.7 (7.8)^d^	.001
Post-PCI FFR, mean (SD)	0.88 (0.07)	0.93 (0.04)^c^^,^^e^	0.84 (0.02)^c^^,^^d^	0.76 (0.04)^d^^,^^e^	<.001
Pre-PCI SYNTAX score, mean (SD)	12.4 (7.4)	11.1 (7.0)^c^^,^^e^	13.5 (7.2)^c^^,^^d^	16.1 (8.5)^d^^,^^e^	<.001
Post-PCI SYNTAX score, mean (SD)	3.7 (5.4)	2.9 (4.7)^c^^,^^e^	4.1 (5.4)^c^^,^^d^	6.9 (7.1)^d^^,^^e^	<.001

Abbreviations: DS, diameter stenosis; FFR, fractional flow reserve; MLD, minimum lumen diameter; PCI, percutaneous coronary intervention; SYNTAX, Synergy Between PCI With Taxus and Cardiac Surgery.

^a^ Unless otherwise indicated, data are expressed as No. (%) of patients.

^b^ The Bonferroni correction was used for pairwise comparisons. The χ^2^ test was used to evaluate associations between categorical variables, and an analysis of variance was performed to compare continuous variables.

^c^ *P* < .05 compared with the residual ischemia group.

^d^ *P* < .05 compared with the optimal group.

^e^ *P* < .05 compared with the suboptimal group.

### Correlation Between Post-PCI FFR and Angiographic Parameters

**Figure 1.**
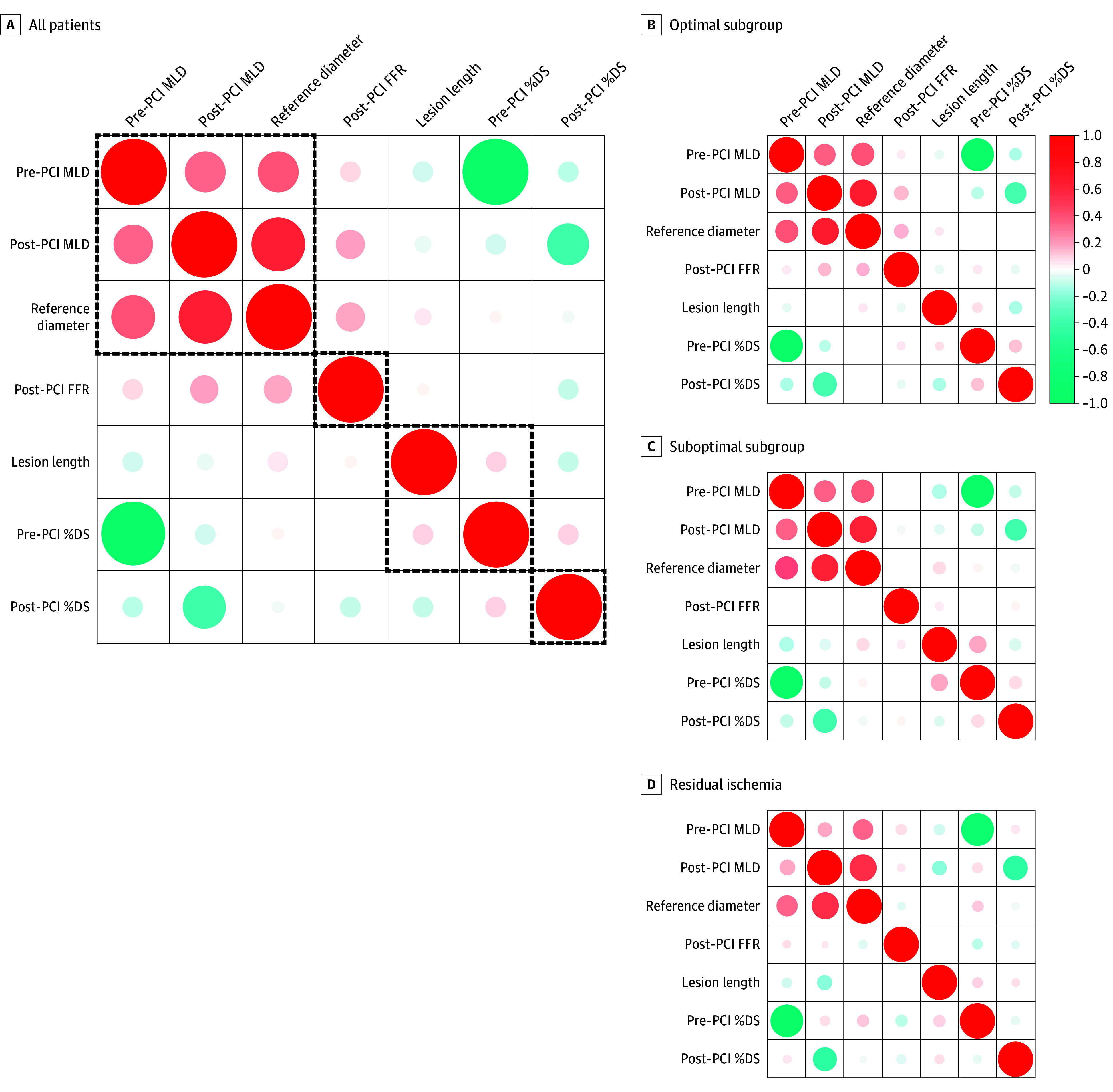
Unsupervised Hierarchical Clustering of Angiographic Parameters and Post–Percutaneous Coronary Intervention (PCI) Fractional Flow Reserve (FFR) According to Post-PCI Physiologic Status Post-PCI FFR was isolated from any angiographic parameters in the unsupervised hierarchical cluster analysis. The size and color of the circles present the degree of correlations, and the dotted black lines denote the cluster from the hierarchical clustering. Hierarchical clustering is one of the algorithms from unsupervised machine learning, which is used to group similar objects. For the optimal subgroup, the correlation coefficient ranges from −1 to 1, where values close to −1 suggest a negative association, values close to 1 indicate a positive association, and values close to 0 indicate no linear association. MLD indicates minimum lumen diameter; %DS, percentage diameter stenosis.

The angiographic parameters and post-PCI FFR showed poor correlations in the total population (lesion length, *r* = 0.03 [*P* = .17]; pre-PCI MLD, *r* = 0.08 [*P* < .001]; post-PCI MLD, *r* = 0.18 [*P* < .001]; and post-PCI percentage diameter stenosis, *r* = −0.10 [*P* < .001]) (eFigure and eTable 1 in Supplement 1). In particular, there was no correlation between pre-PCI percentage diameter stenosis and post-PCI FFR (pre-PCI percentage diameter stenosis, *r* = 0.0003 [*P* = .99]). These correlations were insignificant in the suboptimal group (lesion length, *r* = 0.07 [*P* = .10]; pre-PCI MLD, *r* = −0.04 [*P* = .30]; pre-PCI percentage diameter stenosis, *r* = 0.04 [*P* = .36]; post-PCI MLD, *r* = −0.02 [*P* = .60]; and post-PCI percentage diameter stenosis, *r* = 0.00002 [*P* > .99]) and residual ischemia group (lesion length, *r* = 0.01 [*P* = .85]; pre-PCI MLD, *r* = 0.07 [*P* = .28]; pre-PCI percentage diameter stenosis, *r* = −0.10 [*P* = .10], post-PCI MLD, *r* = 0.04 [*P* = .47]; and post-PCI percentage diameter stenosis, *r* = −0.06 [*P* = .37]) groups, unlike the optimal group (lesion length, *r* = −0.02 [*P* = .58]; pre-PCI MLD, *r* = 0.02 [*P* = .47]; pre-PCI percentage diameter stenosis, *r* = 0.05 [*P* = .05], post-PCI MLD, *r* = 0.16 [*P* < .001]; and post-PCI percentage diameter stenosis, *r* = −0.06 [*P* = .02]) (eFigure and eTable 1 in Supplement 1). The unsupervised hierarchical cluster analysis showed that post-PCI FFR was isolated from any other angiographic parameter, not only in the total population but in each subgroup according to the residual functional disease burden (Figure 1).

### Clinical Outcomes According to Post-PCI Physiologic Status

**Figure 2.**
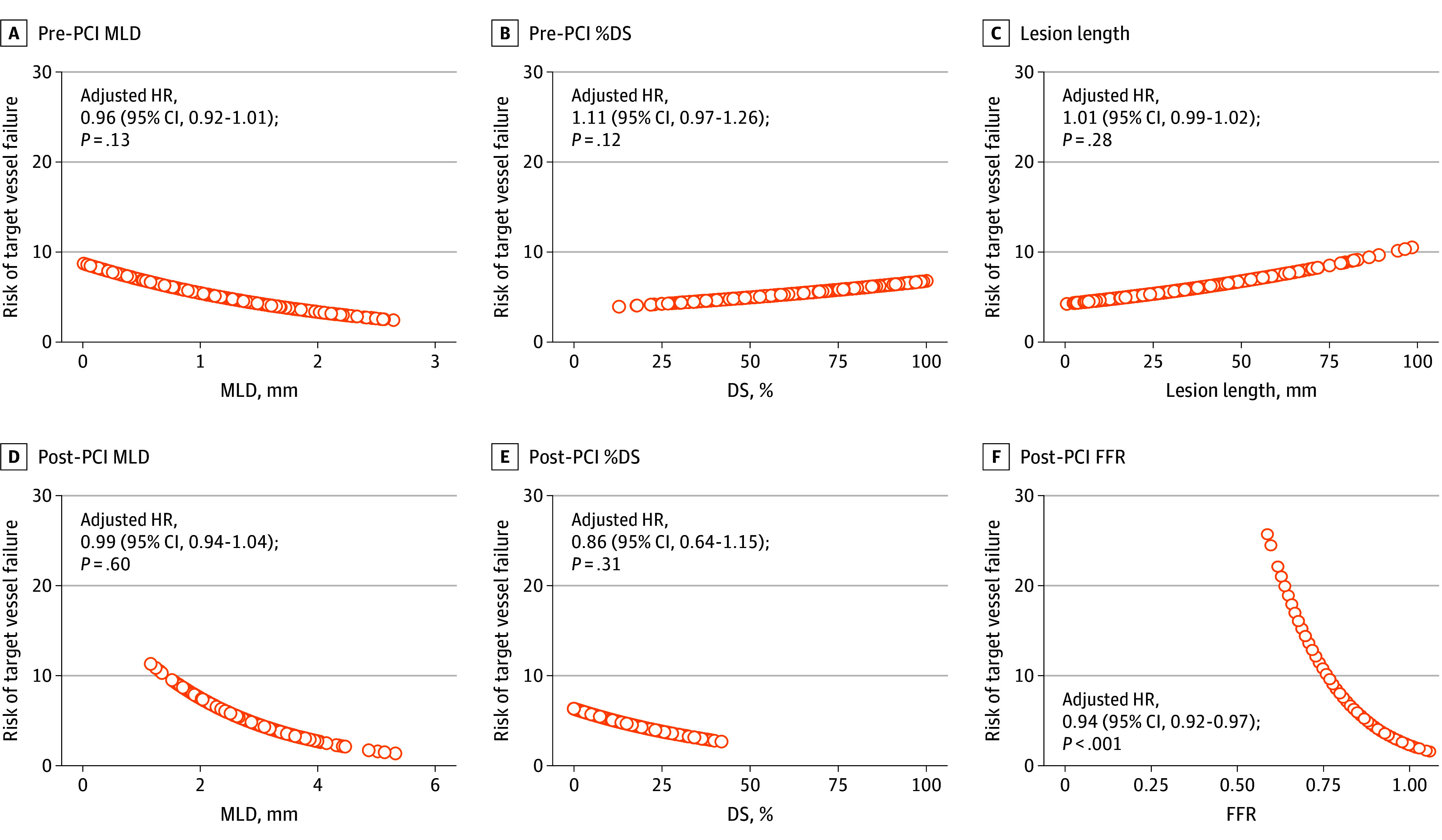
Risk of Target Vessel Failure According to Angiographic and Physiologic Parameters Post–percutaneous coronary intervention (PCI) fractional flow reserve (FFR) was associated with adverse clinical events, but there were no associations between angiographic parameters and any adverse clinical events. The models were adjusted for age, sex, acute coronary syndrome, diabetes, hypertension, left anterior descending artery, and the reference diameter. HR indicates hazard ratio; MLD, minimum lumen diameter; and %DS, percentage diameter stenosis.

**Figure 3.**
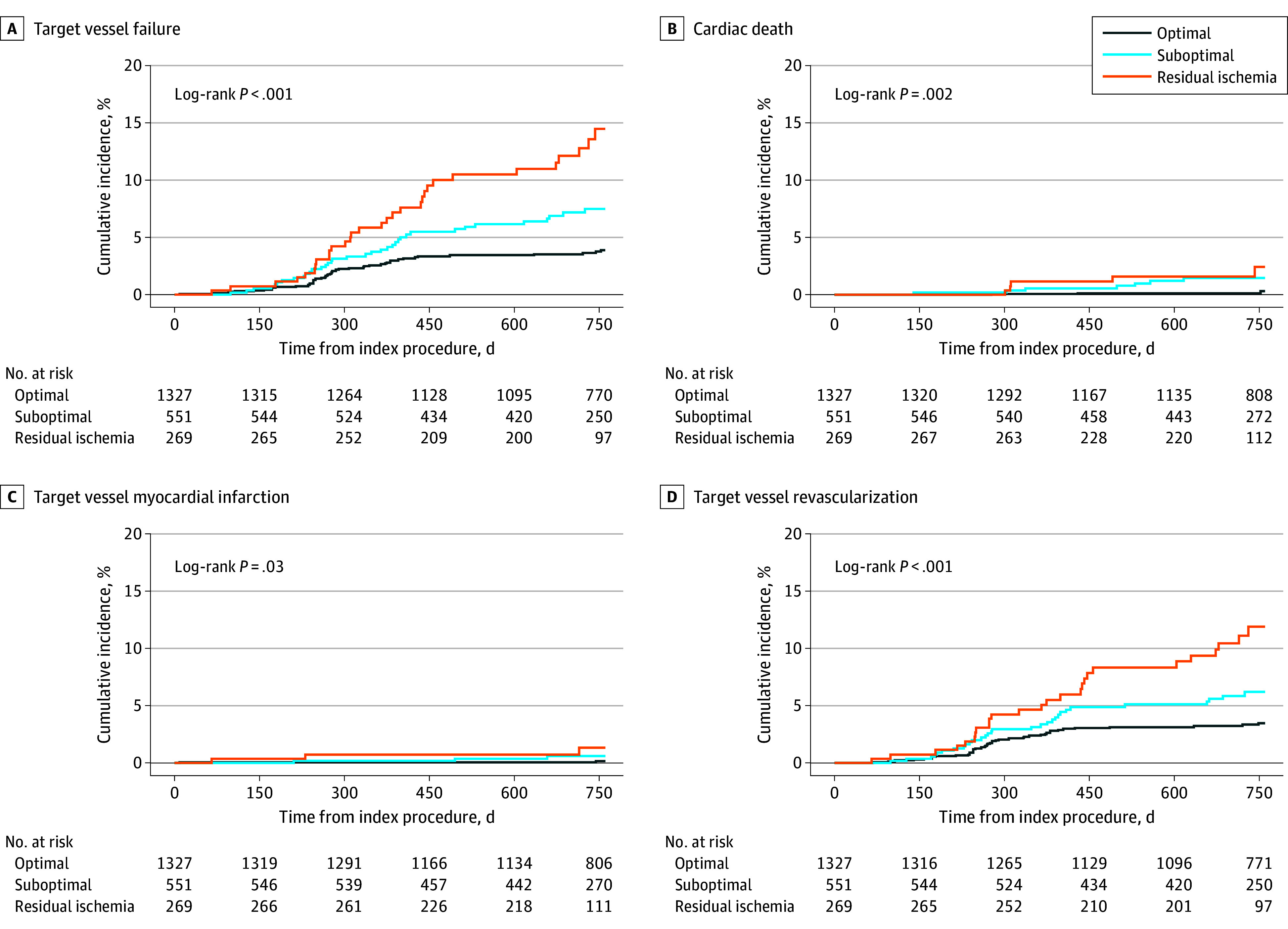
Clinical Events According to Post–Percutaneous Coronary Intervention (PCI) Physiologic Status The risks of target vessel failure and target vessel revascularization increased along with the decrease of post-PCI fractional flow reserve from the optimal PCI group to the suboptimal group and residual ischemia group.

Among angiographic and physiologic parameters after PCI, post-PCI FFR was associated with the risk of TVF (AHR per post-PCI FFR 0.01 increase, 0.94 [95% CI, 0.92-0.97]; *P* < .001) (Figure 2). However, angiographic parameters were not associated with the risk of TVF (Figure 2). These results were consistent regardless of residual functional disease burden and location of revascularization (eTables 2 and 3 in Supplement 1). The cumulative incidences of TVF at 2 years were 3.8% (47 events) in the optimal group, 7.2% (36 events) in the suboptimal group, and 13.5% (31 events) in the residual ischemia groups (*P* < .001) (Figure 3 and eTable 4 in Supplement 1). The risk of TVF was higher in the residual ischemia group than in the suboptimal group (AHR, 1.75 [95% CI, 1.08-2.83]; *P* = .02) and the optimal group (AHR, 2.94 [95% CI, 1.82-4.73]; *P* < .001) (eTable 5 in Supplement 1). In addition, post-PCI FFR itself and post-PCI physiologic status by post-PCI FFR were independently associated with TVF, along with age and clinical presentation as acute coronary syndrome (eTable 6 in Supplement 1).

**Figure 4.**
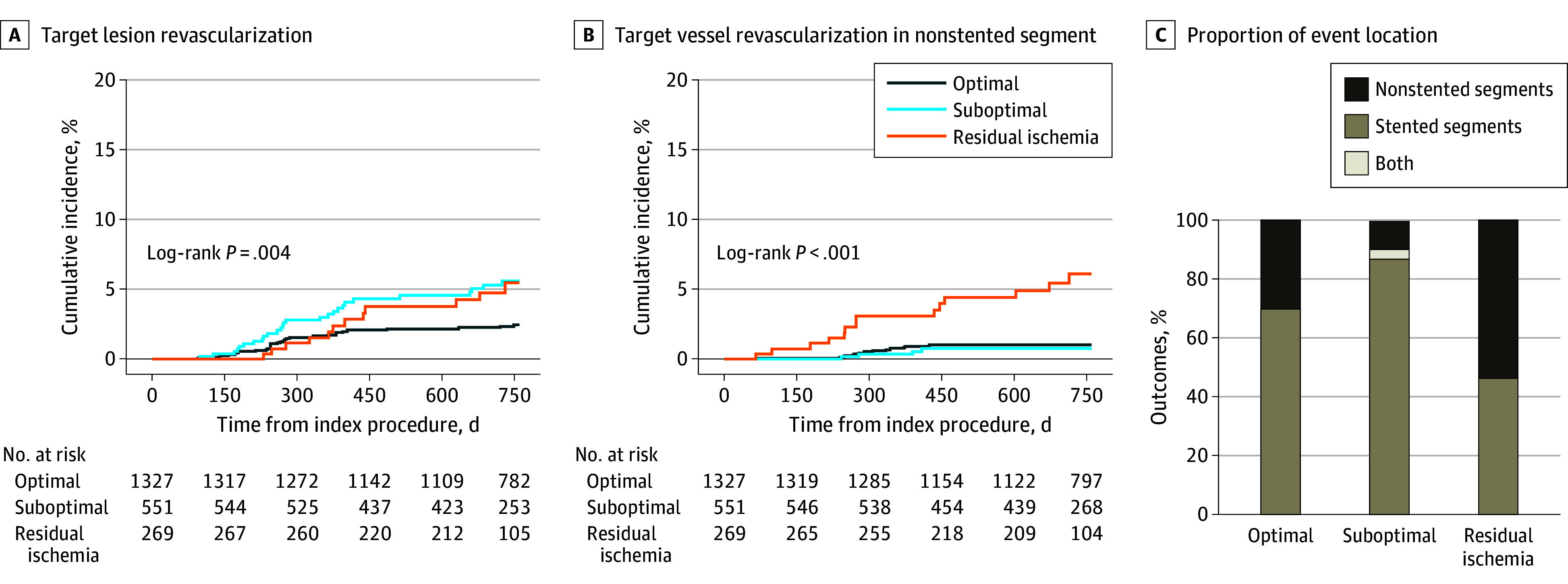
Locations of Events According to Post–Percutaneous Coronary Intervention Physiologic Status The risks of target lesion revascularization (TLR) were comparable between the residual ischemia group and the suboptimal group, and those of target vessel revascularization (TVR) in nonstented segments were comparable between the suboptimal group and the optimal group. Furthermore, more than half of TVRs were in nonstented segment in the residual ischemia group, and most TVRs were TLRs in suboptimal and optimal groups.

Cumulative incidences of TVR at 2 years increased with decreasing post-PCI FFR (eTable 4 in Supplement 1 and Figure 3). The residual ischemia group had a higher risk of TVR than the optimal group (AHR, 2.77 [95% CI, 1.66-4.63]; *P* < .001), and this risk difference was mainly associated with the risk of non–target lesion revascularization (TLR) TVR (eTable 5 in Supplement 1). Although the risk of TVR was also higher in the residual ischemia group than in the suboptimal group (AHR, 1.76 [95% CI, 1.04-2.98]; *P* = .04), the risk of TLR was comparable. The proportions of TLR and non-TLR TVR showed different patterns according to the residual functional disease burden (Figure 4). The TVR in the residual ischemia group was predominantly associated with TVR in the nonstented segment (14 [53.8%]), unlike the other 2 groups (3 [10.0%] in the suboptimal group and 13 [30.2%] in the optimal group). (Figure 4).

## Discussion

This cohort study investigated the association and clinical implication of angiographic parameters and post-PCI FFR according to residual functional disease burden. The major findings were as follows. First, angiographic parameters showed poor correlation with post-PCI FFR, regardless of residual functional disease burden. Post-PCI FFR was isolated from any angiographic parameter in the unsupervised hierarchical cluster analysis. Second, post-PCI FFR was associated with adverse clinical events; however, there were no associations between angiographic parameters and any adverse clinical event. Third, the risk of TVF and TVR decreased along with the increase of post-PCI FFR in the optimal group relative to the suboptimal and residual ischemia groups. More than half of TVRs were non-TLR TVR in the residual ischemia group, and most TVRs were TLR in the post-PCI physiologically suboptimal group and the optimal group.

### Association Between Angiographic Parameters and Post-PCI FFR

In current daily practice, PCI results are primarily evaluated based on findings from invasive coronary angiography, and angiographically successful PCI is often defined as a residual stenosis of less than 20% after PCI.^5^ However, myocardial perfusion imaging studies^25,26^ have demonstrated that residual myocardial ischemia was often present even after successful PCI. Post-PCI FFR can reflect the residual disease burden after PCI originating from stented and nonstented segments in the coronary artery,^6,7^ and previous studies have reported that residual ischemia based on post-PCI FFR was found in 10% to 36% of patients after PCI.^27,28,29,30,31^ In line with these previous studies, 12.5% of patients in the present study had residual ischemia. The correlations between angiographic parameters and post-PCI FFR were poor regardless of residual functional disease burden. Furthermore, in the hierarchical cluster analysis, post-PCI FFR was isolated from any other angiographic parameter. These results indicate the limitations of using post-PCI angiographic parameters to evaluate PCI outcomes. Furthermore, our study showed that none of the angiographic parameters were associated with the risks of TVF or TLR. Unlike previous studies that reported the association between the in-stent percentage diameter stenosis and the risk of TLR, the present study included patients with angiographically successful PCI. This suggests that angiographic parameters are less informative after angiographically successful PCI.^32,33^

### Distributions of Clinical Events According to Post-PCI FFR

Low post-PCI FFR is associated with outcomes in patients with coronary artery disease. The association between the physiologic result after PCI and the risk of clinical events has been fully evaluated by previous studies.^6,7,10,11,13,19,28,29,34,35,36,37,38^ However, most prior studies^13,19,28,34,35,36,37^ have used a binary approach by comparing clinical outcomes using 1 cutoff value of post-PCI FFR. Limited studies with small sample size^39^ have examined post-PCI physiologic status–related outcomes, encompassing both the residual ischemia and optimal result cutoff values concurrently. The current study, which enrolled more than 2000 patients, offers a unique opportunity for evaluating the distributions of individual components of TVF across 3 distinct groups based on the post-PCI physiologic status, including the optimal, suboptimal, and residual ischemia groups. In our study, the residual ischemia group showed not only the highest mortality rate, but also the highest rate of TVR compared with the other 2 groups. Nevertheless, the suboptimal group displayed a higher mortality, yet a similar rate of TVR, compared with the optimal PCI group. These findings highlight potentially different implications between the suboptimal physiologic outcome and residual ischemia.

Since post-PCI FFR can reflect the residual disease burden in stented and nonstented segments of the coronary artery, it is natural that post-PCI FFR can be a surrogate marker for vessel-specific clinical outcomes. Of note, low post-PCI FFR is influenced by the pattern and distribution of coronary disease and is responsible for the poor prognosis.^40,41^ Patients with diffuse disease present suboptimal post-PCI physiologic results more often compared with patients with focal lesions. In patients with long lesions, a satisfactory post-PCI FFR was achieved in only 26.2% of patients, and nearly 20% of patients presented with ischemia at follow-up.^42^ One study^41^ evaluated the clinical implications of functional residual disease patterns using the pressure gradient index and instantaneous quantitative flow ratio gradient per unit length and demonstrated a pattern of diffuse disease, where the major gradient had the highest event rate compared with the other patterns. In terms of the revascularizations, our findings are in line with those of previous observations; specifically, the group with residual ischemia exhibited the highest TVR rate, particularly in nonstented segments. In contrast, the suboptimal and optimal groups demonstrated nondominant rates of non-TLR TVR, and the occurrence of non-TLR TVR was significantly lower in both groups compared with the residual ischemia group. These results suggest that as the post-PCI FFR values decreased, clinical events associated with nonstented segments became more dominant. Moreover, even though post-PCI FFR is only a number reflecting the residual ischemic burden of an epicardial coronary artery, this value can provide information regarding the part of the coronary lesion that will be associated with future adverse events.

### Limitations

This study has several limitations. First, the data from the present study were acquired from previous observational registries; therefore, the inherent limitations of an observational study should be considered. Second, previous studies^40,43^ have demonstrated the clinical importance of physiologic patterns in coronary disease and the transstent pressure gradient. However, the pullback data of post-PCI FFR were not available in the present study. The association of post-PCI FFR with outcomes can be affected by the physiologic patterns in coronary disease or the transstent pressure gradient. Third, the post-PCI FFR values used in the current study were measured after clinically and angiographically successful PCIs. While the present study could not provide information regarding the effects of additional intervention in patients with residual ischemia or suboptimal groups, the results offer insights into the associations between angiographic parameters and post-PCI FFR and its value in routine clinical practice. Further study is warranted to reveal the benefit of further interventions for patients with the suboptimal results of post-PCI FFR. Fourth, the current study included a cohort of relatively low-risk patients, characterized by a mean SYNTAX score of less than 13 (a SYNTAX score below 23 is widely recognized as indicative of low risk). Therefore, extrapolation of the current results should be done cautiously when applied to patients with complex lesions. Furthermore, information regarding medical treatment was not available.

## Conclusions

In this cohort study, a low degree of correlations was observed between angiographic and physiologic parameters after PCI. Post-PCI FFR, unlike angiographic parameters, was associated with clinical events and the distribution of subsequent TVRs. The present study supports the use of post-PCI FFR as a procedural quality metric, and further prospective study is warranted.

## Notes

Supplement 1. **eFigure.** Correlations Between Post-PCI FFR and Angiographic Parameters **eTable 1.** Correlations Between Post-PCI FFR and Angiographic Parameters **eTable 2.** Association Between Angiographic Parameters and the Risk of Target Vessel Failure According to Post-PCI Physiologic Status **eTable 3.** Association Between Post-PCI Angiographic Parameter and Lesion Locations According to Post-PCI Physiologic Status **eTable 4.** Clinical Outcomes According to Post-PCI Physiologic Status **eTable 5.** Comparison of Clinical Outcomes According to Post-PCI Physiologic Status **eTable 6.** Independent Predictors of Target Vessel Failure

Supplement 2.
Data Sharing Statement
