# Supplementary material for: Angiographic Findings and Post–Percutaneous Coronary Intervention Fractional Flow Reserve
Source: JAMA Netw Open. 2024 Jun 21;7(6):e2418072. doi: 10.1001/jamanetworkopen.2024.18072 (PMC11193130; doi:10.1001/jamanetworkopen.2024.18072)
Supplement: Supplement 1. — eFigure. Correlations Between Post-PCI FFR and Angiographic Parameters eTable 1. Correlations Between Post-PCI FFR and Angiographic Parameters eTable 2. Association Between Angiographic Parameters and the Risk of Target Vessel Failure According to Post-PCI Physiologic Status eTable 3. Association Between Post-PCI Angiographic Parameter and Lesion Locations According to Post-PCI Physiologic Status eTable 4. Clinical Outcomes According to Post-PCI Physiologic Status eTable 5. Comparison of Clinical Outcomes According to Post-PCI Physiologic Status eTable 6. Independent Predictors of Target Vessel Failure [file jamanetwopen-e2418072-s001.pdf]

Supplementary Online Content

Zhang J, Hwang D, Yang S, et al. Angiographic and post–percutaneous coronary intervention functional flow reserve parameters. *JAMA Netw Open*. 2024;7(6):e2418072. doi:10.1001/jamanetworkopen.2024.18072

eFigure. Correlations Between Post-PCI FFR and Angiographic Parameters
eTable 1. Correlations Between Post-PCI FFR and Angiographic Parameters
eTable 2. Association Between Angiographic Parameters and the Risk of Target Vessel Failure According to Post-PCI Physiologic Status
eTable 3. Association Between Post-PCI Angiographic Parameter and Lesion Locations According to Post-PCI Physiologic Status
eTable 4. Clinical Outcomes According to Post-PCI Physiologic Status
eTable 5. Comparison of Clinical Outcomes According to Post-PCI Physiologic Status
eTable 6. Independent Predictors of Target Vessel Failure

This supplementary material has been provided by the authors to give readers additional information about their work.

© 2024 Zhang J et al. *JAMA Network Open*.

eFigure. Correlations Between Post-PCI FFR and Angiographic Parameters

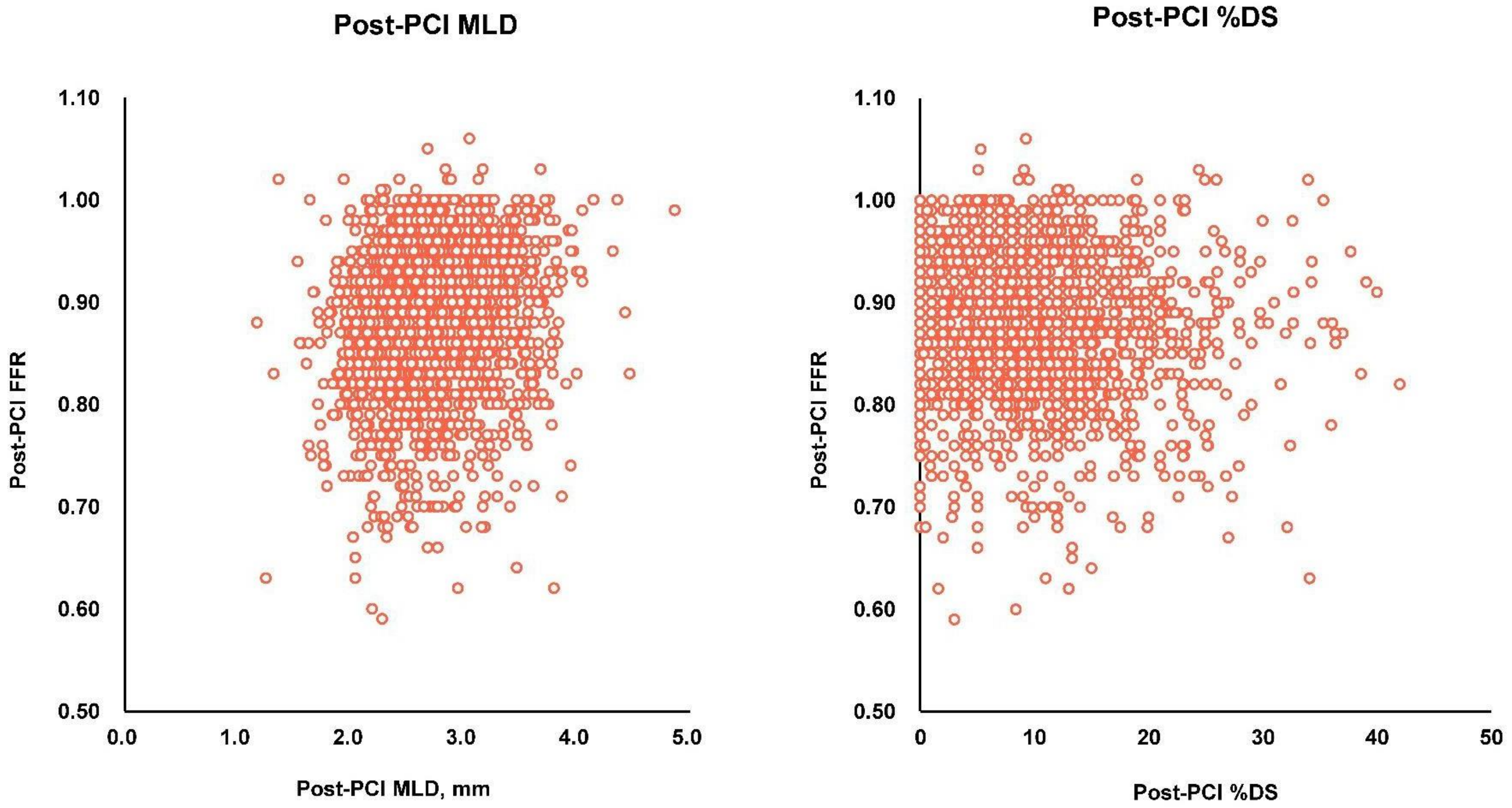

Post-PCI FFR showed poor correlations with the post-PCI angiographic parameters.

Abbreviations: FFR, fractional flow reserve; MLD, minimum lumen diameter; PCI, percutaneous coronary intervention; %DS, percent diameter stenosis.

© 2024 Zhang J et al. *JAMA Network Open*.

eTable 1. Correlations Between Angiographic Parameters and Post-PCI FFR

	All patients		Optimal (n=1,327)		Suboptimal (n=551)		Residual Ischemia (n=269)	
	**r**	***p* value**	**r**	***p* value**	**r**	***p* value**	**r**	***p* value**
Lesion length	0.03	0.17	-0.02	0.58	0.07	0.10	0.01	0.85
Pre-PCI MLD	0.08	<0.001	0.02	0.47	-0.04	0.30	0.07	0.28
Pre-PCI %DS	0.00	0.99	0.05	0.05	0.04	0.36	-0.10	0.10
Post-PCI MLD	0.18	<0.001	0.16	<0.001	-0.02	0.60	0.04	0.47
Post-PCI %DS	-0.10	<0.001	-0.06	0.02	0.00	1.00	-0.06	0.37

Pearson's correlation coefficient was calculated to assess the linear association between variables.

Abbreviations: FFR, fractional flow reserve; MLD, minimum lumen diameter; PCI, percutaneous coronary intervention; %DS, percent diameter stenosis

© 2024 Zhang J et al. *JAMA Network Open*.

eTable 2. Association Between Angiographic Parameters and the Risk of Target Vessel Failure According to Post-PCI Physiologic Status

	Adjusted HR	95% CI	*p* value
Optimal			
Lesion length, per 1mm	1.01	0.99 – 1.03	0.53
Pre-PCI MLD, per 0.1mm	0.95	0.88 – 1.01	0.12
Post-PCI MLD, per 0.1mm	1.02	0.94 – 1.10	0.71
Pre-PCI %DS, per 10%	1.16	0.96 – 1.42	0.13
Post-PCI %DS, per 10%	0.74	0.45 – 1.22	0.24
Suboptimal			
Lesion length, per 1mm	1.02	1.00 – 1.04	0.13
Pre-PCI MLD, per 0.1mm	0.98	0.90 – 1.06	0.58
Post-PCI MLD, per 0.1mm	0.94	0.85 – 1.03	0.19
Pre-PCI %DS, per 10%	1.10	0.87 – 1.38	0.42
Post-PCI %DS, per 10%	0.84	0.50 – 1.41	0.50
Residual ischemia			
Lesion length, per 1mm	0.99	0.97 – 1.02	0.58
Pre-PCI MLD, per 0.1mm	1.01	0.91 – 1.12	0.86
Post-PCI MLD, per 0.1mm	1.02	0.93 – 1.11	0.72
Pre-PCI %DS, per 10%	0.94	072 – 1.24	0.67
Post-PCI %DS, per 10%	0.98	0.62 – 1.56	0.94

The hazard ratios and 95% confidence intervals are calculated by the multivariable cox proportional hazard regression model adjusted by age, sex, acute coronary syndrome, diabetes mellitus, hypertension, left anterior descending artery, and reference diameter.

Abbreviations: %DS, percent diameter stenosis; CI, confidence interval; HR, hazard ratio; MLD, minimal lumen diameter; PCI, percutaneous coronary intervention

© 2024 Zhang J et al. *JAMA Network Open*.

eTable 3. Association Between Post-PCI Angiographic Parameter and Lesion Locations According to Post-PCI Physiologic Status

	Adjusted HR	95% CI	*p* value
Total population			
**Target lesion revascularization**			
Post-PCI MLD, 0.1mm	0.96	0.89 – 1.03	0.21
Post-PCI %DS, 10%	0.95	0.66 – 1.36	0.77
**Non-TLR TVR**			
Post-PCI MLD, 0.1mm	1.03	0.94 – 1.13	0.57
Post-PCI %DS, 10%	0.65	0.36 – 1.18	0.16
Optimal			
**Target lesion revascularization**			
Post-PCI MLD, 0.1mm	1.03	0.93 – 1.14	0.62
Post-PCI %DS, 10%	0.71	0.37 – 1.36	0.30
**Non-TLR TVR**			
Post-PCI MLD, 0.1mm	0.97	0.82 – 1.15	0.72
Post-PCI %DS, 10%	0.64	0.24 – 1.75	0.39
Suboptimal			
**Target lesion revascularization**			
Post-PCI MLD, 0.1mm	0.95	0.84 – 1.06	0.35
Post-PCI %DS, 10%	0.89	0.49 – 1.60	0.69
**Non-TLR TVR**			
Post-PCI MLD, 0.1mm	0.82	0.55 – 1.24	0.35
Post-PCI %DS, 10%	0.27	0.02 – 3.11	0.29
Residual ischemia			
**Target lesion revascularization**			
Post-PCI MLD, 0.1mm	0.85	0.72 – 1.01	0.06
Post-PCI %DS, 10%	1.59	0.79 – 3.20	0.20
**Non-TLR TVR**			
Post-PCI MLD, 0.1mm	1.10	0.98 – 1.24	0.11
Post-PCI %DS, 10%	0.79	0.39 – 1.59	0.51

© 2024 Zhang J et al. *JAMA Network Open*.

The hazard ratios and 95% confidence intervals are calculated by the multivariable cox proportional hazard regression model adjusted by age, sex, acute coronary syndrome, diabetes mellitus, hypertension, left anterior descending artery, and reference diameter.

Abbreviations: CI, confidence interval; HR, hazard ratio; MLD, minimum lumen diameter; non-TLR TVR, non-target lesion revascularization but target vessel revascularization.; PCI, percutaneous coronary intervention; %DS, percent diameter stenosis.

© 2024 Zhang J et al. *JAMA Network Open*.

eTable 4. Clinical Outcomes According to Post-PCI Physiologic Status

	**Optimal (n=1,327) Event number (%)**	**Suboptimal (n=551) Event number (%)**	**Residual ischemia (n=269) Event number (%)**	***p* value**
Target vessel failure	47(3.8)	36(7.2)	31(13.5)	<0.001
Cardiac death	3(0.3)	7(1.4)	5(2.4)	0.002
Target vessel MI	2(0.2)	3(0.6)	3(1.3)	0.03
Target vessel revascularization	43(3.4)	30(6.0)	26(11.2)	<0.001
Target lesion revascularization	30(2.4)	27(5.4)	12(5.3)	0.004
Non-TLR TVR	13(1.0)	4(0.8)	14(5.9)	<0.001

Values are the number of events with their cumulative incidence, n (%). The cumulative incidence was presented based on Kaplan–Meier censoring estimates. A log-rank test was used to compare the cumulative incidence of clinical events.

Abbreviations: MI, myocardial infarction; non-TLR TVR, non-target lesion revascularization but target vessel revascularization; PCI, percutaneous coronary intervention.

© 2024 Zhang J et al. *JAMA Network Open*.

eTable 5. Comparison of Clinical Outcomes According to Post-PCI Physiologic Status

	Compared to the optimal group			
	**Residual ischemia group**		**Suboptimal group**	
	**Adjusted HR (95% CI)**		**Adjusted HR (95% CI)**	
Target vessel failure	2.94 (1.82 – 4.73)	<0.001	1.68 (1.07 – 2.65)	0.02
Cardiac death	7.95 (1.76 – 35.79)	0.007	5.11 (1.25 – 20.96)	0.02
Target vessel MI	6.13 (0.94 – 40.08)	0.058	3.18 (0.50 – 20.34)	0.22
Target vessel revascularization	2.77 (1.66 – 4.63)	<0.001	1.57 (0.97 – 2.56)	0.07
Target lesion revascularization	2.07 (1.20 – 3.57)	0.009	1.86 (0.93 – 3.73)	0.08
Non-TLR TVR	4.47 (1.98 – 10.05)	<0.001	0.66 (0.21 – 2.08)	0.48

The hazard ratios and 95% confidence intervals are calculated by the multivariable cox proportional hazard regression model adjusted by age, sex, acute coronary syndrome, diabetes mellitus, hypertension, left anterior descending artery, and reference diameter.

Abbreviations: CI, confidence interval; HR, hazard ratio; MI, myocardial infarction; non-TLR TVR, non-target lesion revascularization but target vessel revascularization; PCI, percutaneous coronary intervention.

© 2024 Zhang J et al. *JAMA Network Open*.

eTable 6. Independent Predictors of Target Vessel Failure

	Adjusted HR	95% CI	*p* value
Age, per 1 year	1.02	1.00 – 1.04	0.02
Sex (female as reference)	1.36	0.84 – 2.20	0.21
Acute coronary syndrome	1.51	1.04 – 2.21	0.03
Diabetes mellitus	1.46	1.00 – 2.13	0.05
Hypertension	1.10	0.73 – 1.66	0.65
Left anterior descending artery	1.23	0.76 – 1.98	0.40
Post-PCI FFR, per 0.01	0.94	0.92 – 0.96	<0.001
Age, per 1 year	1.02	1.00 – 1.04	0.03
Sex (female as reference)	1.34	0.83 – 2.17	0.23
Acute coronary syndrome	1.49	1.02 – 2.17	0.04
Diabetes mellitus	1.44	0.99 – 2.11	0.06
Hypertension	1.07	0.71 – 1.62	0.74
Left anterior descending artery	1.33	0.82 – 2.15	0.25
Post-PCI physiologic status			
Optimal (reference)	-	-	-
Suboptimal	1.81	1.15 – 2.84	0.01
Residual ischemia	3.40	2.09 – 5.55	< 0.001

Abbreviations: CI, confidence interval; FFR, fractional flow reserve; HR, hazard ratio; PCI, percutaneous coronary intervention.

© 2024 Zhang J et al. *JAMA Network Open*.
