# Supplementary material for: Angiographic Findings and Post–Percutaneous Coronary Intervention Fractional Flow Reserve
Source: JAMA Netw Open. 2024 Jun 21;7(6):e2418072. doi: 10.1001/jamanetworkopen.2024.18072 (PMC11193130; doi:10.1001/jamanetworkopen.2024.18072)
Supplement: Supplement 2. — Data Sharing Statement [file jamanetwopen-e2418072-s002.pdf]

Data Sharing Statement

Zhang. Angiographic Findings and Post–Percutaneous Coronary Intervention Fractional Flow Reserve. *JAMA Netw Open*. Published June 21, 2024.
doi:10.1001/jamanetworkopen.2024.18072

Data
Data available: No
